# An interpretable approach for automatic aesthetic assessment of remote sensing images

**DOI:** 10.3389/fncom.2022.1077439

**Published:** 2022-11-24

**Authors:** Jingru Tong, Guo Zhang, Peijie Kong, Yu Rao, Zhengkai Wei, Hao Cui, Qing Guan

**Affiliations:** ^1^School of Remote Sensing and Information Engineering, Wuhan University, Wuhan, China; ^2^State Key Laboratory of Information Engineering in Surveying, Mapping, and Remote Sensing, Wuhan University, Wuhan, China

**Keywords:** remote sensing images, aesthetic assessment, aesthetic quality, interpretability, attention mechanism, deep learning

## Abstract

The increase of remote sensing images in recent decades has resulted in their use in non-scientific fields such as environmental protection, education, and art. In this situation, we need to focus on the aesthetic assessment of remote sensing, which has received little attention in research. While according to studies on human brain’s attention mechanism, certain areas of an image can trigger visual stimuli during aesthetic evaluation. Inspired by this, we used convolutional neural network (CNN), a deep learning model resembling the human neural system, for image aesthetic assessment. So we propose an interpretable approach for automatic aesthetic assessment of remote sensing images. Firstly, we created the Remote Sensing Aesthetics Dataset (RSAD). We collected remote sensing images from Google Earth, designed the four evaluation criteria of remote sensing image aesthetic quality—color harmony, light and shadow, prominent theme, and visual balance—and then labeled the samples based on expert photographers’ judgment on the four evaluation criteria. Secondly, we feed RSAD into the ResNet-18 architecture for training. Experimental results show that the proposed method can accurately identify visually pleasing remote sensing images. Finally, we provided a visual explanation of aesthetic assessment by adopting Gradient-weighted Class Activation Mapping (Grad-CAM) to highlight the important image area that influenced model’s decision. Overall, this paper is the first to propose and realize automatic aesthetic assessment of remote sensing images, contributing to the non-scientific applications of remote sensing and demonstrating the interpretability of deep-learning based image aesthetic evaluation.

## Introduction

In recent decades, remote sensing has advanced rapidly, becoming increasingly important in geological mapping, environmental monitoring, urban development, etc. These studies mainly focus on the scientific uses of remote sensing. However, with the increase of remote sensing images, they have emerged in various non-scientific applications and been used non-scientific users. These individuals only regard remote sensing images as images, not as a source of scientific information. In such case, we need to pay attention to the aesthetic assessment of remote sensing images. Visually appealing remote sensing images, which offer a distinctive perspective from above, can be meaningful to fields such as environmental protection, education, and art. When policymakers are exposed to natural splendors, they may be motivated to adopt more environmentally friendly measures (Wang et al., 2016). When creating artworks, artists such as photographers and painters can be inspired by the beauty of the Earth (Grayson, 2016). Beautiful remote sensing images can also be used by educators to trigger students’ passion in nature. According to studies on human brain’s attention mechanism, certain areas of an image can trigger visual stimuli, influencing aesthetic evaluation. Inspired by this, we used convolutional neural network (CNN), a deep learning model resembling the human neural system, to perform automatic aesthetic assessment of remote sensing images. By comparing the key image area that affected the model’s decision with human aesthetic standards, we discussed the interpretability of deep-learning based image aesthetic evaluation.

Aesthetic assessment is the process of classifying images into high or low aesthetic quality (Wong and Low, 2009; Luo et al., 2011), or predict their aesthetic scores (Datta and Wang, 2010; Li et al., 2010). Aesthetic quality can be understood as the pleasure people obtain from appreciating images (Kalivoda et al., 2014). Recent advances in cognitive neuroscience have suggested correspondence between the physical properties of stimuli and the sensations they cause (Skov and Nadal, 2020). Therefore, images of high aesthetic quality can be deemed as “visually pleasing.” Though people’s aesthetic preference or criteria may differ (Kim et al., 2018), such subjectivity does not preclude objective research into aesthetic quality. Just as many people may feel more comfortable and delightful with certain rhythms in music (Li and Chen, 2009), many may have similar feelings towards certain images. The same goes for remote sensing images. And if we can identify the factors that affect people’s judgment on the aesthetic quality of remote sensing images, we may establish the evaluation standards behind aesthetic evaluation. Using data-driven methods, we can then measure the aesthetic quality of remote sensing images in a scientific way.

In past decades, researchers have designed handcrafted features to quantify image aesthetic quality. These features range from low-level image statistics, such as edge distributions and color histograms, to high-level photographic rules, such as the rule of thirds and the golden ratio. For example, Datta et al. (2006) designed a set of visual features, including color metrics, rule of thirds, depth of field, etc. Using professional photography techniques Luo and Tang (2008) first extracted the subject region from a photo and then formulated many high-level semantic features based on this subject and background division. Recently, researchers began to apply deep learning in image aesthetic evaluation. They typically cast it as a classification or regression problem (Deng et al., 2017). A model is trained by assigning a single label (i.e., a class or score) to an image to indicate its level of aesthetic quality. Compared with hand-crafted features designed primarily based on domain-specific knowledge, automatically learned deep features can better capture the underlying aesthetic characteristics from massive training images (Tian et al., 2015). Among the deep learning methods, CNN proved to be effective in analyzing image aesthetics. It is the most similar to human visual processing systems, has a structure well-suited to processing 2D and 3D images, and can effectively learn and extract 2D feature abstractions. The max-pooling layers of CNN can effectively detect shape changes. And it is good at extracting mid-to-high level abstract features from raw images by interleaving convolutional and pooling layers (i.e., by spatially shrinking feature maps layer by layer).

Here, we tackle the aesthetic assessment problem by binary classification, discriminating a remote sensing image into “high aesthetic quality” or “low aesthetic quality.” And CNNs have excellent performance in image aesthetic classification. In Lu et al. (2014), proposed the Rating Pictorial Aesthetics using Deep Learning (RAPID) model, it was the first attempt to apply CNNs in image aesthetic evaluation. The network structure was close to AlexNet and aimed at the binary aesthetic classification. CNN’s robustness in image aesthetic classification is also demonstrated in image style classification (Karayev et al., 2013) and image popularity estimation (Khosla et al., 2014). In image classification, network depth is crucial, but stacking more conventional layers to increase depth can easily lead to the problem of gradient explosion (Liu et al., 2019). Existing CNN networks, such as AlexNet and VGG, are usually built to directly learn the mapping between input and output, which can hardly alleviate gradient explosion. To address this problem, He et al. (2016) proposed ResNet in 2016, which used residual blocks to create a shortcut between the target and the input. The ResNet residual module can solve the problem of vanishing gradients and accelerate training (Wu et al., 2020).

Despite the good performance of deep neural networks in image aesthetic assessment, they are hard to interpret because they cannot be decomposed into intuitive and understandable components (Lipton, 2018). Evidence from human perception process (Mnih et al., 2014) demonstrates the importance of attention mechanism, which uses top information to guide bottom-up feed-forward process. In the cognitive process of visual aesthetics, the region of the object’s prominent visual properties, such as color, shape, and composition, receives initial attention (Cela-Conde et al., 2011). These prominent regions would trigger stimulus within the ventral visual stream. The feed-forward process would then enhance the visual experience of the object, leading to aesthetic assessment. In other words, rather than processing the whole scene in its entirety, humans selectively focus on specific parts of the image (Wang et al., 2018). So inspired by such attention mechanism involved in image aesthetic evaluation, we adopt the Gradient-weighted Class Activation Mapping (Grad-CAM) proposed by Selvaraju et al. (2017). Grad-CAM can use the gradient information learned by convolutional neurons to highlight the important image area that influenced the model’s decision. The highlighted area generated by Grad-CAM is comparable to the prominent area that draws attention and triggers visual stimulus during the cognitive process of aesthetic assessment.

The increase of remote sensing images in recent decades has resulted in their use by non-scientific users who only see them as images rather than a source of scientific information. In this situation, we need to focus on the aesthetic assessment of remote sensing, which has received little attention in research. Though convolutional neural network (CNN) performs well in image aesthetic evaluation, it lacks interpretability. While according to studies on human brain’s attention mechanism, certain areas of an image can trigger visual stimuli during aesthetic evaluation. Therefore, inspired by the brain’s cognitive process and the use of CNN in image aesthetic assessment, we propose an interpretable approach for automatic aesthetic assessment of remote sensing images. Firstly, we created the Remote Sensing Aesthetics Dataset (RSAD). We collected remote sensing images from Google Earth, designed the four evaluation criteria of remote sensing image aesthetic quality—color harmony, light and shadow, prominent theme, and visual balance—and then labeled the samples based on expert photographers’ judgment on the four evaluation criteria. Secondly, we feed RSAD into the ResNet-18 architecture for training. Experimental results show that the proposed method can accurately identify visually pleasing remote sensing images. Finally, we provided a visual explanation of aesthetic assessment by adopting Grad-CAM to highlight the important image area that influenced model’s decision. Overall, this paper is the first to propose and realize automatic aesthetic assessment of remote sensing images, contributing to the non-scientific applications of remote sensing and demonstrating the interpretability of image aesthetics. Our work has the potential to promote the use of remote sensing in non-scientific fields such as environmental protection, education, and art.

## Materials and methods

**FIGURE 1:**
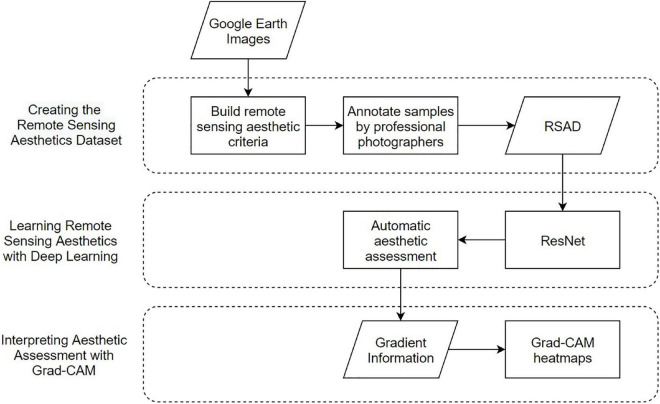
Overall technical route of automatic remote sensing aesthetic assessment.

Our method consists of three steps, as shown in Figure 1. We first created the Remote Sensing Aesthetics Dataset. We collected remote sensing images from Google Earth, established four evaluation criteria of remote sensing aesthetics, and labeled the images based on professional photographers’ judgment of the four criteria. Secondly, we fed the dataset into a deep learning model to classify remote sensing images in high or low aesthetic quality. Finally, we tried to interpret model’s aesthetic assessment with Grad-CAM.

### The remote sensing aesthetics dataset

#### Data source

**FIGURE 2:**
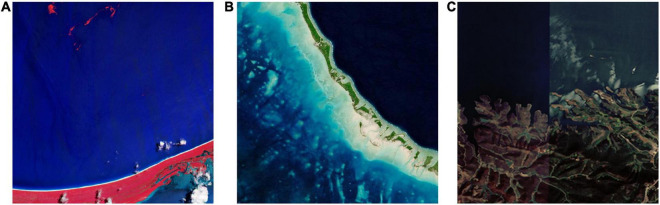
Remote sensing image in false color **(A)**, true color **(B)**, and with artifacts **(C)**.

To enable aesthetic evaluation, the remote sensing images we gather should adhere to certain technical requirements. First, all images should be in true color. They should be combination of the three channels that are sensitive to the red, green, and blue visible light, producing what our naked eyes see in the natural world. As we will explain in the following subsection, color plays a significant role in aesthetic evaluation, and dealing colors we are familiar with is a good place to start when exploring remote sensing aesthetics. Figure 2 compares remote sensing image in true color (Figure 2B) with false color (Figure 2A). Second, samples ought to have a high resolution. In this way, people can identify features on the image and determine whether the image have a prominent theme or visual weight. Finally, images should not contain any artifacts. Artifacts can appear during image mosaicking as a result of color differences or geometric misalignments between adjacent images (Yin et al., 2022), as shown in Figure 2C.

To meet the following technical requirements, we collected images from Google Earth, an open-source platform that includes data integration of satellite and aerial images. Both image types can be regarded as remote sensing images because they are passively collected remotely sensed data. Google Earth includes a wide range of true-color visible spectrum imagery (380–760 nm wavelength) derived from a combination of freely available public domain Landsat imagery, government orthophotos, and high resolution commercial data sets from DigitalGlobe, GeoEye, and SPOT (Fisher et al., 2012). Whatever imaging modalities are used for different data sources, these images all truly reflect the earth’s surface. Also, Google image has a resolution of below 100 m, usually 30 m, and a viewing angle of about 15 km above sea level. As a result, Google Earth images can be used as a data source for assessing remote sensing aesthetic quality.

In order for an effective and thorough investigation of remote sensing aesthetics, we should ensure that the dataset had enough variety. Therefore, we gathered remote sensing images covering eight content categories: river, mountain, farmland, beach, desert, forest, glacier, and plain. These categories are based on typical landscape types and remote sensing features, and they are selected for two reasons. First, these are natural features. These images are simpler and clearer than those with artificial features such as airport, industrial, and residential regions, making it relatively easier for aesthetics quality evaluation. Second, these features are common on the Earth’s surface. They contain a variety of spatial patterns that are representative in terms of texture and color, and most of them vary sufficiently between different regions. For instance, Mount Himalayan, Sahara Desert volcanoes, and frost-covered Arctic mountains are located at different latitudes, and they look completely different.

**FIGURE 3:**
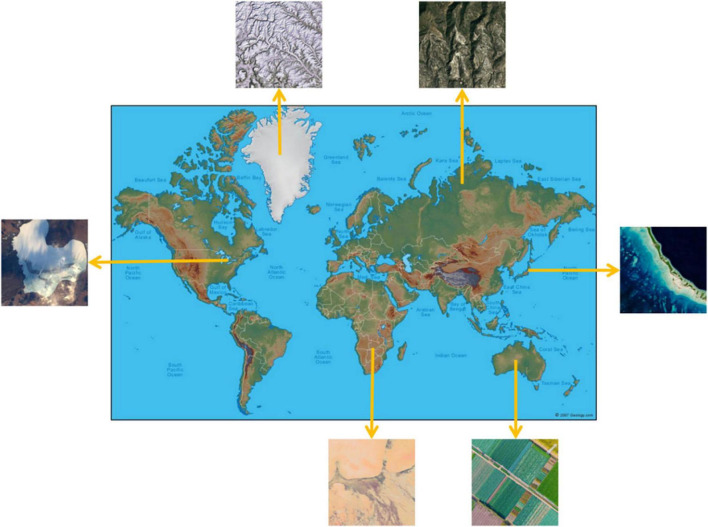
Schematic diagram of remote sensing aesthetics dataset (RSAD) images and their selected locations.

We collected all images from a viewing height of 1,500 m, and we avoided images with artifacts. In addition, to increase diversity, remote sensing images are carefully selected from continents worldwide, covering as many latitudes and regions as possible. And these images are selected from different years and seasons. Figure 3 is a schematic diagram of some images and their selected locations.

#### Evaluation criteria of remote sensing aesthetic quality

Researchers found that image aesthetic quality can be affected by numerous factors, including lighting (Freeman, 2007), contrast (Itten, 1975), color scheme (Shamoi et al., 2020), and image composition (London et al., 2011), etc. While judging the aesthetic quality of remote sensing images, viewers also have certain criteria or pay attention to certain features in mind. Therefore, we first design a questionnaire to study the factors that may influence how humans evaluate the aesthetic quality of remote sensing images.

We recruited a total of 30 college students between the ages of 18 and 25 as volunteers to fill in the questionnaires. To ensure variety, these students come from a variety of backgrounds and major in fields including journalism, law, economics, computer science, psychology, and electrical engineering, etc. There is a nearly equal distribution of genders. In the questionnaire, we presented volunteers with several remote sensing images and asked them to list more than two factors that they felt crucial for assessing the aesthetic quality of these images. They were also encouraged to further explain how the factor affected the aesthetic evaluation. The top four frequently mentioned factors are “Composition,” “Color,” “Content,” and “Light/Brightness.” Other factors mentioned include “Texture,” “Balance,” “Imagination,” “Perspective,” “Mood,” etc.

In response to the survey results, we summarized four evaluation criteria: color harmony, light and shadow, prominent theme, and visual balance, which addressed both the image’s content and composition. As was previously stated, the bottom-up attention mechanism involved in aesthetic evaluation is stimulus-driven. Thus, these four criteria together work as visual stimuli that draw viewers’ attention. In our work, we assume that remote sensing images of high aesthetic quality are used for non-scientific users. These individuals regard remote sensing images solely as images, or in a broader sense, artworks of nature. Therefore, when concluding the aforementioned criteria, we considered the general guidelines for both art and photography. We also considered the properties of remote sensing images. The four criteria are elaborated as follows.

##### Color harmony

**FIGURE 4:**
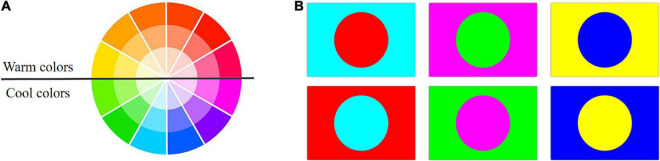
Cool-warm colors **(A)** and complementary colors **(B)**.

Color is what we notice first when we appreciate an image. When two or more colors are brought together to produce a satisfying affective response, they are said to be harmonized (Burchett, 2002). Color harmony is therefore related to the relationship between colors, including cool-warm colors, complementary colors, and the arrangement relations of colors, as shown in Figure 4. A remote sensing image can cover a wide range of features, and the various colors of these features can result in color harmony, leading to high aesthetic quality. The illustration and examples of color harmony in different contexts are provided below.

**FIGURE 5:**
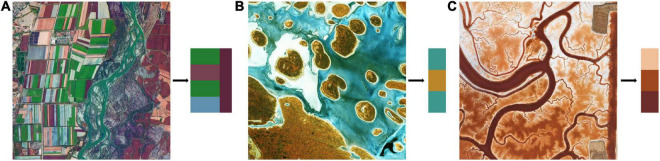
Remote sensing images with cool-warm contrast **(A)**, red-green color complementation **(B)**, and progressive color arrangement **(C)**.

In modern color theories, an imaginary dividing line running through the color wheel separates the colors into warm and cool, as is displayed in Figure 4A. Cool-warm colors are linked to the feelings they evoke and the emotions with which we identify when looking at them. Red, orange, and yellow are warm colors, while blue, green, and purple are cool (Moreland, 2009). Figure 5A cleverly combines cool and warm colors. Cool colors like green and blue predominate in the farmland on the left portion of the image, while warm red predominates in the right portion. They form an overall structure of cool-warm contrast. Meanwhile, the left part is interspersed by warm red color patches, creating a local contrast between cool and warm. Complementary colors are a pair of color stimuli (dependent on appropriate wavelength pairs and luminance ratios) whose mixture color matches a given neutral (Brill, 2007). These color pairs can create a striking visual impact when they appear in the same picture. According to the RGB additive color mode, red and cyan, green and magenta, blue, and yellow are typical complementary color pairs, as is shown by Figure 4B. Remote sensing images that capture complementary color pairs in nature can have a strong visual impact on the audience, resulting in a high aesthetic quality. Figure 5B is an excellent example of red-green complementation, with scattered red islands dotting the green salty lake, bringing liveliness to the whole scene. When colors are arranged in certain relations, they engage the viewer and create an inner sense of order, a balance in the visual experience (Brady and Phillips, 2003). One typical of color arrangement relations is that colors of similar hues undergo progressive changes in brightness or saturation. The gradual change in color will serve as a one-way visual guide, leading humans to appreciate the scene in a specific direction. The progressive red color transition can be seen in the meandering river in Figure 5C.

##### Light and shadow

**FIGURE 6:**
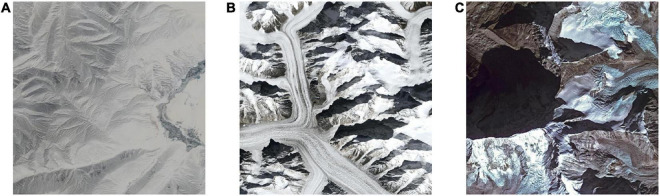
Remote sensing images with a lack of sunlight **(A)**, ideal light-shadow distribution **(B)**, and too large shadow area **(C)**.

Optical remote sensing images, in most cases, use sunlight as a source of illumination (Yamazaki et al., 2009). When sunlight reaches the ground features, it will cast a shadow. A right proportion of light and shade can impart depth perception to the scene, creating a stereoscopic effect (Todd et al., 1997). The amount of shadow produced by the light is determined by its direction. In remote sensing images, the direction of light depends on the solar zenith angle, which is related to the latitude of the direct solar point, the local latitude, and the local time (Zhang et al., 2021). In the morning or afternoon, due to the low solar zenith angle, half of the feature is in sunlight and the other half is in shadow. At this time, the contrast between the bright and dark portions of the image is sharp, and the stereoscopic effect at its peak. However, the ground features’ large shadow area lowers the aesthetic quality at the same time. At noon, the solar zenith angle is close to 90 degrees, so the ground features are evenly exposed to light and can be clearly identified. But the shadow is also the shortest, and the stereoscopic effect is weak. Remote sensing images of high aesthetic quality should have a light-shadow balance. Figure 6B shows an ideal light-shadow distribution that results in high aesthetic quality. The right amount of shadow is produced with enough light and the right light direction: just enough to create the stereoscopic effect without shading over other features. While Figure 6A suffers from the lack of sunlight which results in a dim image, the light direction in Figure 6C creates too large shadow area that obscures the ice in the image, lowering the overall aesthetic quality.

##### Prominent theme

**FIGURE 7:**
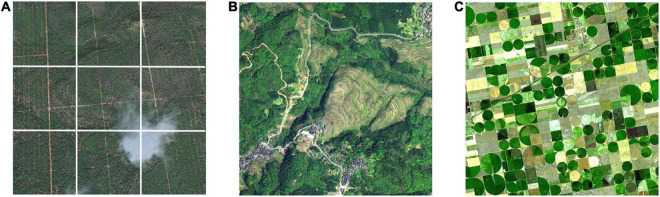
Remote sensing images that emphasize the theme using the rule of thirds **(A)**, framing **(B)**, and repetition **(C)**.

Since remote sensing images are taken from high altitudes, they are often occupied by dense ground features, which can easily make the viewer feel monotonous because of the lack of focus. Therefore, remote sensing image of high aesthetic quality should highlight the theme, drawing the viewer’s attention to the key area of the picture. And the theme is often emphasized by image composition (Dhar et al., 2011), including rule of thirds, framing and repetition. When composing an image, professional photographers often divide the image using the imagery horizontal and vertical thirds lines and place important objects along these lines or at their intersections. This particular visual element placement is known as the rule of thirds (Krages, 2005). In Figure 7A, for example, the heart-shaped cloud is located at the intersection of two dividing lines. The cloud becomes a standout theme, with the green terrain serving as the backdrop. Just as the frame of a painting naturally draws people’s attention to the painting, the frame of an object within an image does the same. A frame can be regular, complete, and closed, or it can be irregular, incomplete, and open. In Figure 7B, dark green woodlands, winding roads and houses form a frame to surround and highlight the colorful terraces. Apart from traditional image composition techniques, repetition can also be used to create a prominent theme. Repetition means using repeating shapes or a repetitive pattern inside the frame as part of the composition. While the overall repetition can easily draw attention and deepen the viewer’s memory of the repeated objects, the repetitions that are slightly different from each other can produce a unique sense of rhythm in the picture (Shinkle, 2004). In remote sensing images, repetitive objects can be seen everywhere. From the bird’s-eye view, these repetitive objects appear as different regular geometric figures, highlighting the distinct theme. Figure 7C serves as a good example. The repetitive circles in different shades of green, which are dotted with rectangle fields of corn and wheat, emphasize the image theme of farmlands.

##### Visual balance

**FIGURE 8:**
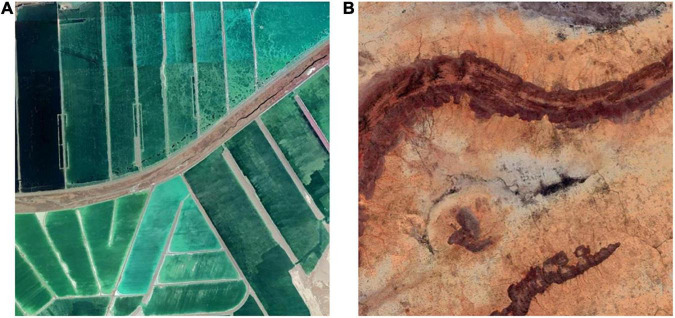
Object area **(A)** and distance from the image center **(B)** impact visual balance.

Visual balance, a sense of weighted clarity created in a composition (Arnheim, 1956), influences how we perceive aesthetic quality (Palmer et al., 2013). Visual balance builds upon the notion of visual weight, a perceptual analog to physical weight (Lok et al., 2004). An object is visually heavy if it takes up large area. The larger the area occupied by an object, the greater its visual weight is. Also, objects far from the image center frequently appear visually heavier than objects close to the image center. This is the visual Principle of Lever: Since the feature in the image represents a heavy object and the image center represents the lever’s fulcrum, the distance between them functions as a lever (Xia, 2020). Figure 8A shows how object area impacts visual balance. The top and bottom portions of the image divided by a tilted line are roughly the same size, whereas the two parts at the bottom are almost equally sized and are divided by a second, nearly diagonal line. Figure 8B shows how distance from the image center impacts visual balance. The long ridge on the upper part of the remote sensing image is of high visual weight. However, such visual weight is balanced by a smaller ridge farther from the center.

#### Dataset creation

**FIGURE 9:**
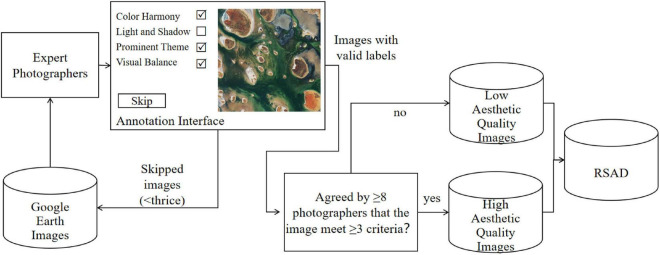
The overall labeling procedure of remote sensing aesthetics dataset (RSAD).

After that, images are manually annotated. We invited professional photographers to evaluate the aesthetic quality of remote sensing images because they master photographic skills and understands the aesthetic preference of the public. They can decide whether the image satisfies the four evaluation criteria: color harmony, light and shadow, prominent theme and visual balance. If a photographer thinks an image satisfies at least three standards, the image will be considered beautiful. 15 photographers participated in the labeling procedure. If 8 or more photographers agree on the aesthetic quality of an image, then we will assign it the label of “high aesthetic quality”. And the remaining images will be of “low aesthetic quality”. In addition, we have added a “skip” option. To put it another way, if the photographer is unable to determine whether a remote sensing image satisfies the four standards, he can skip it. After three skips, an image’s aesthetic quality is suggested to be blurred, so it will be removed from the dataset. The overall annotation process is depicted in Figure 9.

**FIGURE 10:**
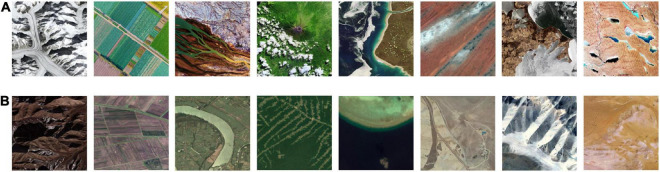
Remote sensing aesthetics dataset (RSAD) samples of high **(A)** and low **(B)** aesthetic quality.

The expert photographers evaluated 1,500 samples, 117 of which were skipped, leaving 1,383 samples with valid labels. The RSAD dataset was finished with 875 positive samples and 508 negative samples. Figure 10 depicts samples of high (A) and low (B) aesthetic quality; images in the same column are of the same content type.

### Learning remote sensing aesthetics with deep learning

In this study, we used binary classification to discriminate a remote sensing image into “high aesthetic quality” or “low aesthetic quality.” And ResNet-18 served as the backbone network. The ResNet residual module can solve the problem of vanishing gradients and is calculated as follows. Define a residual block in the form of y_l_=h(X_l_) + F(X_l_,W_L_), where x and y are the input and output vectors of the residual block, respectively, h(X_l_) is the feature mapping function, and F(X_l_,W_L_) is the residual mapping function to be learned, f(y_l_) is the activation function.


(1) $$y_{l}=h\,\left(X_{l}\right)+F\,\left(X_{l},W_{L}\right)$$

(2) $$X_{\text{l}+1}=f\;\left(y_{l}\right)$$

**FIGURE 11:**
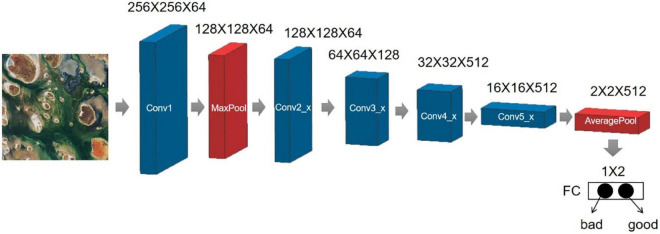
The ResNet-18 network structure and parameters.

Figure 11 depicts the ResNet-18 network structure and parameters, including the input, output, and convolutional and pooling layers in the middle. Input images of 512 x 512 and get the output of 1 x 2 after training. The first parameter represents the probability of being unaesthetic, and the second digit is the probability of being aesthetic. If the probability of being aesthetic is greater than the probability of not being aesthetic, the image is considered visually appealing, and vice versa.

The input section consists of a large convolution kernel (7 x 7, stride 2) and a max-pooling (3 x 3, stride 2). This step converts the 512 x 512 input image to a 128 x 128 feature map. The convolution layer then extracts feature information using two 3 x 3 convolutions and adds it directly to the original data in a residual block; the output part converts the feature map to 1 x 1 using global adaptive average pooling and passes it through the fully connected layer. Table 1 displays the model’s input and output for each layer.

**TABLE 1:** ResNet-18’s input and output for each layer.

Layer name	Operation	Input	Output
Conv1	7 × 7, 64, stride2	512 × 512 × 1	256 × 265 × 64
Max pool	3 × 3, max_pooling, stride2	256 × 265 × 64	128 × 128 × 64
Conv2_x	$\left[\begin{array}{cc} 3\;\times 3 & 64\\ 3\;\times 3 & 64 \end{array}\right]\;\times \;2$	128 × 128 × 64	128 × 128 × 64
Conv3_x	$\left[\begin{array}{cc} 3\;\times 3 & 128\\ 3\;\times 3 & 128 \end{array}\right]\;\times \;2$	128 × 128 × 64	64 × 64 × 128
Conv4_x	$\left[\begin{array}{cc} 3\;\times 3 & 256\\ 3\;\times 3 & 256 \end{array}\right]\;\times \;2$	64 × 64 × 128	32 × 32 × 256
Conv5_x	$\left[\begin{array}{cc} 3\;\times 3 & 512\\ 3\;\times 3 & 512 \end{array}\right]\;\times \;2$	32 × 32 × 256	16 × 16 × 512
Average pool	avg_pooling	16 × 16 × 512	2 × 2 × 512
FC	1,000–d fc + softmax	2 × 2 × 512	1 × 2

### Interpreting aesthetic assessment with gradient-weighted class activation mapping

While deep learning enables good performance in the aesthetic classification of remote sensing images, it lacks interpretability. As the process of aesthetic evaluation involves visual stimulation (Cheung et al., 2019), visualizing the prominent image area that influenced model’s decision can be a solution. Therefore, in an effort to interpret the deep-learning based aesthetic assessment and compare it with the cognitive process of human brain, we adopted the class activation map Grad-CAM proposed by Selvaraju et al. (2017). By referring to the gradient information learned by convolutional neurons, we can generate visual explanations from any CNN-based network without architectural changes or retraining.

**FIGURE 12:**
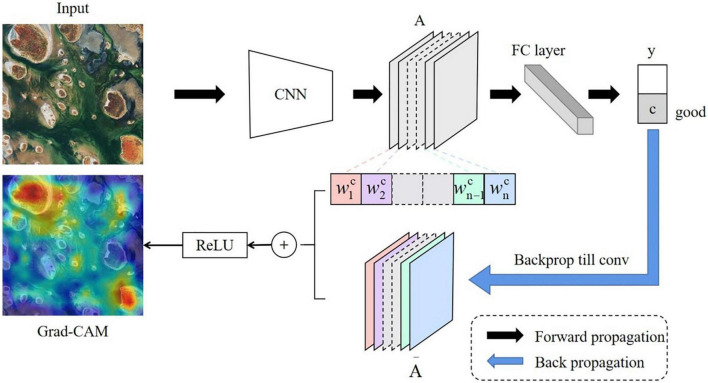
The work flow of gradient-weighted class activation mapping (Grad-CAM).

Gradient-weighted class activation mapping (Grad-CAM) uses the gradient information flowing into the last convolutional layer to draw a heat map, as shown in Figure 12. The network first performs forward propagation to obtain the output of feature layer A (the last convolutional layer of ResNet in this case) and the predicted value y. Assuming that the predicted value of a remote sensing image by the network is y*^c^*, then back-propagating y*^c^* can obtain the gradient information $\overset{-}{A}$ that is back-transmitted to the feature layer. The importance of each channel of the feature layer A is obtained by calculation and then weighted and summed. After passing through the residual module ReLU, we can obtained the final result of Grad-CAM.

Equations 3 and 4 show the Grad-CAM calculation formula. Among them, $A_{\text{ij}}^{k}$ represents the point (i, j) of the kth channel of feature map A, *y*^c^ represents the output of class c, and $\frac{\partial y^{\text{c}}}{\partial A_{i\,j}^{k}}$ represents the partial derivative of *y*^c^ for all feature maps $A_{\text{ij}}^{k}$ of the last layer of CNN. The ReLU function produces a heat map whose values are positively correlated with class c. The negative part indicates that it does not belong to class c, which can be viewed as posing an inhibitory effect and thus can be filtered out with ReLU.


(3) $$\alpha_{k}^{c}=\frac{1}{Z}\,\sum_{i}\sum_{j}\frac{\partial y^{c}}{\partial A_{i\,j}^{k}}$$

(4) $$L_{G\,r\,a\,d-C\,A\,M}^{c}=R\,e\,L\,U\;\left(\sum_{k}\alpha_{k}^{c}\,A^{k}\right)$$

The Grad-CAM heat map can show which area contributes the most to an image’s aesthetic quality prediction. The redder parts of the heat map have a greater impact on the prediction than the bluer parts. As a result, using Grad-CAM, we can verify the four evaluation criteria we have concluded of remote sensing image aesthetic quality: color harmony, light and shadow, prominent theme and visual balance.

## Experimental results and analysis

### Experimental design

In this paper, we conducted experiments on the Remote Sensing Aesthetics Dataset. 80% of the samples are for training, and the remaining 20% are for testing. To facilitate network training, we resized the images to 512 x 512 and fed them into the ResNet-18 architecture. After that, we used quantitative indicators to assess model performance.

Regarding training parameters, we trained 100 epochs with ResNet-18, batch size = 16, without any pre-trained weights. Stochastic gradient descent is the optimizer used in back-propagation, with the hyperparameter learning rate set to 1 × 10^–4^. The learning rate controls the update of the weights, and a lower learning rate allows the model to converge better. Cross-entropy is the loss function, and it is defined as follows: *y*_i_ represents the aesthetic label of sample i, the positive class is 1, and the negative class is 0; *p*_i_ represents the probability that sample i is predicted to be a positive class.


(5) $$L=\frac{1}{N}\,\sum_{i}L_{i}=\frac{1}{N}\,\sum_{i}-\left[y_{i}\cdot \log\left(p_{i}\right)+\left(1-y_{i}\right)\cdot \log\left(1-p_{i}\right)\right]$$

### Evaluation metrics

In this paper, finding visually attractive remote sensing images is a binary classification task in which samples are classified as either high or low aesthetic quality. The confusion matrix is thus used to calculate the four parameters TP, FP, TN, and FN to evaluate model performance. Each parameter in the confusion matrix is explained as follows.

• TP (True Positive): High-aesthetic-quality image predicted of high aesthetic quality.
• TN (True Negative): Low-aesthetic-quality image predicted of low aesthetic quality.
• FP (False Positive): Low-aesthetic-quality image predicted of high aesthetic quality.
• FN (False Negative): High-aesthetic-quality image predicted of low aesthetic quality.

We can calculate accuracy, recall, precision, and F1-score of our method based on these four parameters, shown in Equations 6–9.

• Accuracy: the proportion of accurately predicted images in all images.

(6) $$\begin{array}{c} A\,\text{cc}\,u\,r\,a\,c\,y=\frac{\left(T\,P+T\,N\right)}{\left(T\,P+F\,N+T\,N+F\,P\right)}\times 100 \end{array}$$

• Recall: the proportion of accurately predicted aesthetic images in all correct predictions.

(7) $$R\,e\,c\,a\,l\,l=\frac{T\,P}{T\,P\;+\;F\,N}$$

• Precision: the proportion of images predicted as high aesthetic quality of all aesthetic images.

(8) $$P\,\text{re}\,c\,i\,s\,i\,o\,n=\frac{T\,P}{T\,P\;+\;F\,P}$$

• F1-score: the harmonic mean of precision and recall, reflecting the robustness of our model.

(9) $$F\,1=\frac{2\,T\,P}{2\,T\,P\;+\;F\,N\;+\;F\,P}=\frac{2\cdot \mathrm{Pr}e\,c\,i\,s\,i\,o\,n\cdot R\,e\,c\,a\,l\,l}{\mathrm{Pr}e\,c\,i\,s\,i\,o\,n\;+\;R\,e\,c\,a\,l\,l}$$

### Results and analysis

#### Automatic remote sensing aesthetic assessment

**FIGURE 13:**
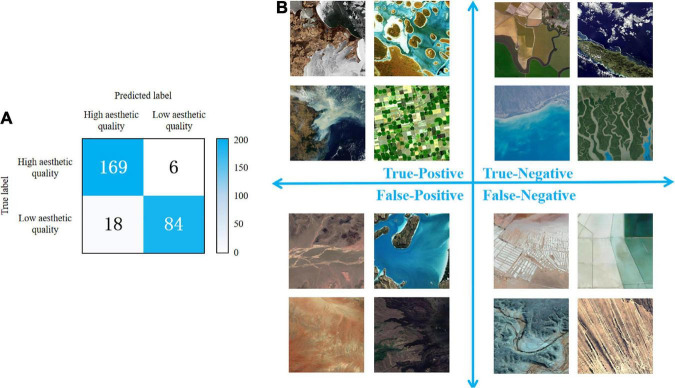
Confusion matrix for the test set **(A)** and the classification results for each cell of the confusion matrix **(B)**.

The test set contains 277 samples, and it has an accuracy of 91.34%. Figure 13A depicts the confusion matrix for the test set, and the classification results for each cell of the confusion matrix are visualized in Figure 13B.

Judging from the True-Positive cell where images of high aesthetic quality are correctly predicted, we can conclude that our model can distinguish the images that meet the four evaluation standards. In the lower-right image of farmland, there is feature repetition and a prominent theme. Light and shadow contrast can be found in the upper-left image of glacier. And color harmony exists in the upper-right image of coral reef. Similar conclusion can be reached when we examine all images in the True-Negative cell. Looking at the farmland image in the upper-left corner with a meandering purple outline and the image of meandering rivers in the lower-right corner, we can see that the model may find the winding shape visually unappealing.

Based on the confusion matrix, we calculated accuracy, recall, precision and F1-score. The accuracy is 91.34%, demonstrating the overall good performance. The precision is 0.90, which indicates the effectiveness of the model in identifying images of low aesthetic quality. Meanwhile, the model is good at identifying high-aesthetic-quality images, as the recall reaches 0.67. While F1-score of 0.77 proves the robustness of the model as well.

From the analysis above, we can conclude that the ResNet model we trained can accurately distinguish between remote sensing images of high and low aesthetic quality.

#### Attention mechanism in automatic aesthetic assessment

**FIGURE 14:**
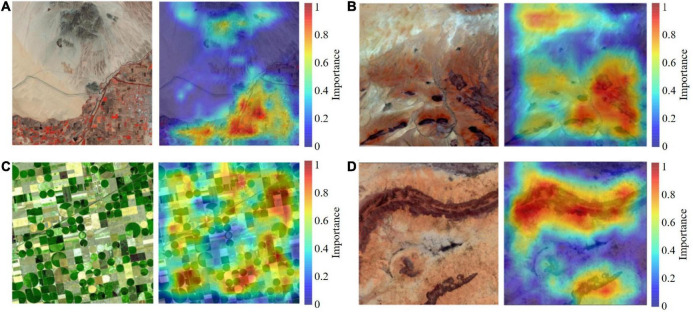
Gradient-weighted class activation mapping (Grad-CAM) captures the image’s cool-warm contrast **(A)**, the light and shadow area **(B)**, the repetitive circles and the rectangle fields of corn and wheat **(C)**, and the two balancing portions of visual weight **(D)**.

In an effort to interpret the deep-learning based aesthetic assessment, we adopted Grad-CAM to highlight the prominent image area that influenced model’s decision, as shown in Figure 14. By examining how those areas matches human attention on the four aesthetic standards, we can compare how ResNet performs aesthetic evaluation with the actual cognitive process of aesthetics in the human brain.

#### Color harmony

Color harmony is related to the relationship between colors, including cool-warm colors, complementary colors and the arrangement relations of colors. Cool-warm colors are linked to the feelings they evoke and the emotions with which we identify when looking at them. Complementary colors are a pair of color stimuli whose mixture color matches a given neutral. And color arrangement relations are the progressive color changes in hue, brightness or saturation. Grad-CAM highlighted the warm red blocks in the lower right corner and the cool-toned mountains in Figure 14A, indicating the cool-warm color contrast.

#### Light and shadow

When ground features are exposed to sunlight, shadows will occur. A right proportion of light and shade can impart depth perception to the scene, creating a stereoscopic effect. However, a large shadow area will reduce the aesthetic quality. So remote sensing image of high aesthetic quality should have light-shadow balance, as shown in Figure 14B. The lower-right corner of the image has more shade areas whereas the upper-left corner has more exposure to light, both are highlighted on the heat map.

#### Prominent theme

Remote sensing image of high aesthetic quality should highlight the theme, drawing the viewer’s attention to the key area of the picture. And prominent theme is realized by repetition, rule of thirds and framing. Figure 14C serves as a good example of repetition. Grad-CAM captures the repetitive circles in various shades of green, as well as the rectangle fields of corn and wheat, all of which emphasize the image theme of farmlands.

#### Visual balance

Visual balance, a sense of weighted clarity created in a composition, is influenced by the feature’s area and its distance from the image center. In Figure 14D, the long ridge on the upper part of the remote sensing image is of high visual weight. A smaller ridge farther from the center, however, balances such visual weight. And both ridges are highlighted on the heat map.

Judging from the heat maps’ highlighted regions, we can conclude that ResNet’s aesthetic evaluation is involved with something similar to the attention mechanism of the brain’s visual aesthetic process. It proves the interpretability of automatic remote sensing aesthetic assessment as well.

## Conclusion and future work

To enable non-scientific application of remote sensing images, while inspired by the brain’s cognitive process and the use of CNN in image aesthetic assessment, we propose an interpretable approach for automatic aesthetic assessment of remote sensing images. Firstly, we created the Remote Sensing Aesthetics Dataset. We collected remote sensing images from Google Earth, designed the four evaluation criteria of remote sensing image aesthetic quality—color harmony, light and shadow, prominent theme, and visual balance—and then labeled the samples based on expert photographers’ judgment on the four evaluation criteria. Secondly, we feed RSAD into the ResNet-18 architecture for training. Experimental results show that the proposed method can accurately identify visually pleasing remote sensing images. Finally, we provided a visual explanation of aesthetic assessment by adopting Grad-CAM to highlight the important image area that influenced model’s decision. Overall, this paper is the first to propose and realize automatic aesthetic assessment of remote sensing images, contributing to the non-scientific applications of remote sensing and demonstrating the interpretability of deep-learning based image aesthetic evaluation.

But some limitations still exist, so we need to further our research. First, we treat aesthetic assessment as a binary classification problem in this paper. This is because assigning an aesthetic quality score requires more voters and samples. Therefore, estimating an aesthetic quality score for each remote sensing image using regression methods will be part of the future work. Second, we only used ResNet, a scene-based CNN, as the backbone of evaluation, which is not a novel method. To ensure that the model is more dedicated to remote sensing aesthetic quality, we should fine-tune the backbone network by adjusting its blocks and layers. Third, objectivity and subjectivity coexist in aesthetic assessment. So we are unable to verify the aesthetic classification results due to the possible subjectivity of aesthetics. Thus, we will continue to work on bridging the objective and subjective aspects of remote sensing aesthetics through well-designed psychology surveys. To sum up, more research and practice in the fields of neural science, remote sensing, deep learning, aesthetics, and psychology will be needed in the future for the automatic aesthetic evaluation of remote sensing images.

## Data availability statement

The original contributions presented in this study are included in the article/supplementary material, further inquiries can be directed to the corresponding author.

## Author contributions

JT: conceptualization, methodology, formal analysis, writing—original draft, and writing—review and editing. GZ: conceptualization and supervision. PK: validation and writing—review and editing. YR: validation and visualization. ZW: investigation and data curation. HC: resources and writing—review and editing. QG: investigation and writing—review and editing. All authors contributed to the article and approved the submitted version.
